# Supplementary material for: Investigation of the Expression Pattern and Functional Role of miR-10b in Intestinal Inflammation
Source: Animals (Basel). 2023 Apr 2;13(7):1236. doi: 10.3390/ani13071236 (PMC10093392; doi:10.3390/ani13071236)
Supplement: Supplementary file 1 [file animals-13-01236-s001.zip › Supplementary Figure S1.pdf]

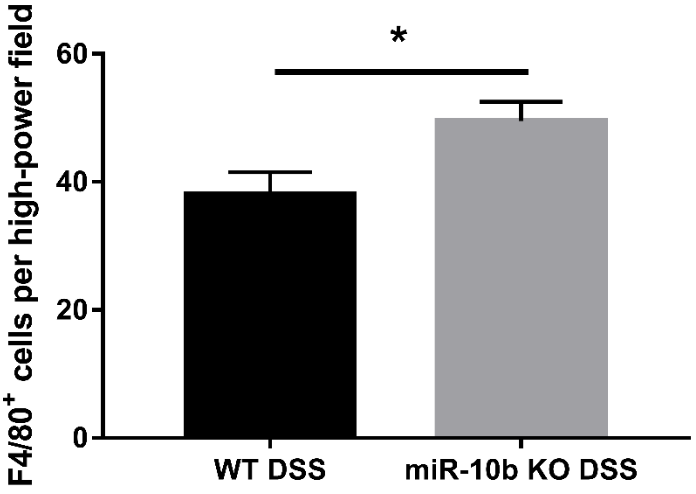

Supplementary Figure S1. Number of F4/80$^+$ macrophages in colonic tissue. The mean number of F4/80$^+$ cells detected in each high-power field was calculated by counting four fields from each sample (samples from three mice per group were counted) (WT, wild-type; KO, knockout; n = 12/group; *, $P < 0.05$).
