# Supplementary material for: Investigation of the Expression Pattern and Functional Role of miR-10b in Intestinal Inflammation
Source: Animals (Basel). 2023 Apr 2;13(7):1236. doi: 10.3390/ani13071236 (PMC10093392; doi:10.3390/ani13071236)
Supplement: Supplementary file 1 [file animals-13-01236-s001.zip › Supplementary Table S1-DAI.pdf]

Table S1. Disease activity index (DAI) scoring system for mice with colitis

Score	Body weight loss	Fecal hardness	Bleeding scores
0	none	normal	none
1	1-5%		
2	6-10%	loose	slight bleeding
3	11-20%		
4	>20%	diarrhea	gross bleeding
