# Supplementary material for: Investigation of the Expression Pattern and Functional Role of miR-10b in Intestinal Inflammation
Source: Animals (Basel). 2023 Apr 2;13(7):1236. doi: 10.3390/ani13071236 (PMC10093392; doi:10.3390/ani13071236)
Supplement: Supplementary file 1 [file animals-13-01236-s001.zip › Supplementary Table S2.pdf]

Table S2. Histological scoring system for mice with colitis

Score	inflammatory cells infiltration	cecal tissue damage
0	normal	normal
1	scattered inflammatory cells in the lamina propria	minimal inflammation and crypt hyperplasia
2	increased numbers of inflammatory cells in the lamina propria	mild crypt hyperplasia
3	confluence of inflammatory cells extending into the submucosa	obvious crypt hyperplasia, invasion of epithelium
4	transmural extension of the infiltrative inflammatory cells	extensive mucosal damage and extension through deeper structures of the bowel wall
