# Supplementary material for: Investigation of the Expression Pattern and Functional Role of miR-10b in Intestinal Inflammation
Source: Animals (Basel). 2023 Apr 2;13(7):1236. doi: 10.3390/ani13071236 (PMC10093392; doi:10.3390/ani13071236)
Supplement: Supplementary file 1 [file animals-13-01236-s001.zip › Supplementary Table S3.pdf]

Table S3. Summary of trimming and read mapping results of the sequences generated from DSS-treated mice

Sample	Raw reads	Clean reads	Q30 (%)	GC content (%)	Total mapped	Multiple mapped	Unique mapped
DSS-KO-1	52080674	51531546	93.42	49.72	49858181(96.75%)	4509170(8.75%)	45349011(88.0%)
DSS-KO-2	46377730	45930040	93.29	49.8	42513831(92.56%)	3736299(8.13%)	38777532(84.43%)
DSS-KO-3	53050710	52481006	93.43	49.55	50763306(96.73%)	5186260(9.88%)	45577046(86.84%)
DSS-KO-4	47379846	46869908	93.43	48.69	45076582(96.17%)	5264493(11.23%)	39812089(84.94%)
DSS-WT-1	52031128	51522142	93.63	49.64	49750406(96.56%)	4919033(9.55%)	44831373(87.01%)
DSS-WT-2	53668680	53147182	93.6	50.08	51461477(96.83%)	4583893(8.62%)	46877584(88.2%)
DSS-WT-3	48003972	47512952	93.44	49.69	45910219(96.63%)	4366966(9.19%)	41543253(87.44%)
DSS-WT-4	51247716	50687948	93.34	50.27	48875594(96.42%)	4263531(8.41%)	44612063(88.01%)
